# An NTCP Analysis of Urethral Complications from Low Doserate Mono- and Bi-Radionuclide Brachytherapy

**DOI:** 10.1155/2011/128360

**Published:** 2011-07-06

**Authors:** V. E. Nuttens, A. E. Nahum, S. Lucas

**Affiliations:** ^1^NAmur Research Institute for LIfe Sciences (NARILIS), Research Center for the Physics of Matter and Radiation (PMR-LARN), University of Namur (FUNDP), Rue de Bruxelles, 61, 5000 Namur, Belgium; ^2^Department of Physics, Clatterbridge Center for Oncology, Clatterbridge Road Bebington, Merseyside CH63 4JY, UK

## Abstract

Urethral NTCP has been determined for three prostates implanted with seeds based on ^125^I (145 Gy), ^103^Pd (125 Gy), ^131^Cs (115 Gy), ^103^Pd-^125^I (145 Gy), or ^103^Pd-^131^Cs (115 Gy or 130 Gy). First, DU_20_, meaning that 20% of the urhral volume receive a dose of at least DU_20_, is converted into an I-125 LDR equivalent DU_20_ in order to use the urethral NTCP model. Second, the propagation of uncertainties through the steps in the NTCP calculation was assessed in order to identify the parameters responsible for large data uncertainties. Two sets of radiobiological parameters were studied. The NTCP results all fall in the 19%–23% range and are associated with large uncertainties, making the comparison difficult. Depending on the dataset chosen, the ranking of NTCP values among the six seed implants studied changes. Moreover, the large uncertainties on the fitting parameters of the urethral NTCP model result in large uncertainty on the NTCP value. In conclusion, the use of NTCP model for permanent brachytherapy is feasible but it is essential that the uncertainties on the parameters in the model be reduced.

## 1. Introduction

Radiotherapy treatment planning systems incorporating “biological” models are beginning to make their way into clinical use. The biological models in questions are for Tumor Control Probability (TCP) [3, 4] and Normal Tissue Complication Probability (NTCP) [5, 6]. A common suggestion is that treatment plans be optimized to maximize the TCP while not exceeding a fixed, acceptable NTCP [7, 8]. A considerable amount of work has been done to implement this biological model-based approach in permanent seed prostate brachytherapy [1, 9–12]. Prostate brachytherapy morbidity is generally reported for the urethra [13–17] and rectum [18–21]. Zaider et al. have extended radiotherapy NTCP models for these organs to Low Dose Rate (LDR) permanent brachytherapy [1, 12]. 

Three radionuclides are generally used in permanent prostate brachytherapy: iodine-125, palladium-103, and cesium-131. Each radionuclide has its advantages but also its drawbacks. ^125^I and ^131^Cs have similar emission spectrum whose mean energies are 28.37 and 30.45 keV, respectively. ^103^Pd has a mean emitted energy of 20.74 keV which reduces the dose to the surrounding organs at risk (OAR) such as rectum and urethra but also increases the risk of cold spots (underdosage) in the prostate. By contrast, the ^125^I and ^131^Cs dose distributions extend to larger distances, thus reducing the likelihood of cold spots at the cost of delivering more to the OARs for a given prostate dose. Urethral and rectal complications are reported at a similar frequency for ^125^I or ^131^Cs [22]. 

The contrast between the properties of ^103^Pd, ^125^I, and ^131^Cs has motivated our research in developing a new kind of seed based on a mixture of two radionuclides, namely, ^103^Pd_0.75_-^125^I_0.25_ or ^103^Pd_0.25_-^131^Cs_0.75_. The subscripts denote the fractions of internal activity of each radionuclide as defined in a previous paper [23]. To avoid a cumbersome style, the ^103^Pd_0.75_-^125^I_0.25_ and ^103^Pd_0.25_-^131^Cs_0.75_ mixture will be referred to in the text as Pd-I and Pd-Cs. The dosimetry characteristics and prescription doses of these sources were derived in previous studies [23, 24]. 

In our study, we use the Zaider et al. NTCP model for the urethra to compare a monoradionuclide seed implant (^103^Pd, ^125^I or ^131^Cs) and bi-radionuclide seed implants (Pd-I or Pd-Cs). A sensitivity analysis on the modeling parameters is also performed.

## 2. Patients and Methods

### 2.1. Treatment Plans

A source data file for the Prostate Seed Implant Dosimetry (PSID) Treatment Planning System (TPS) has been generated for InterSource seeds loaded with either ^103^Pd, ^125^I, ^131^Cs, Pd-I, or Pd-Cs following the AAPM TG43U1 formalism, which we have adapted for multiple radionuclide brachytherapy sources [23]. 

Three patients were planned using peripheral seed placement: patient 1 with the smallest prostate, patient 2 with a medium-sized prostate, and patient 3 with the largest prostate. Prostate and urethra volumes of each patient are given in Table 1. The urethral volume is defined as the volume enclosed by the urethra surface. Each patient has been planned with the five types of seeds. The prescription doses used for bi-radionuclide implants have been derived previously [24] and are summarized in Table 2. The method that we used yields a single, fixed value of 142 Gy for the prescription dose of a Pd-I implant whereas it results in two different values for the Pd-Cs mixture: 115 Gy and 128 Gy. However, these values are associated with large uncertainties. They were rounded to 145 Gy, 115 Gy, and 130 Gy, respectively, as safety margins on tumor control. The total number of treatment plans is therefore 18 (6 plans per patient). 

### 2.2. Urethra NTCP

To the best of our knowledge, only Zaider et al. [1] have developed a model for the urethra NTCP after I-125 LDR. This model is based on a correlation between the probability of urethral toxicity and the dose received by the “hottest” 20% of the urethral volume (DU_20_) after permanent prostate brachytherapy using ^125^I seeds. In order to apply such a model, and because in our study we are dealing with different radionuclides, we have converted the dose distribution for each implant to an equivalent I-125 LDR one. In order to do this, we derive the DU_20-I_ of an I-125 LDR treatment that would yield the same Biologically Effective Dose (BED) as the implant in question. The BED includes a linear and a quadratic term in dose. In the case of bi-radionuclide implant, its formulation requires the contribution of each radionuclide to the DU_20_. The models and procedure are described in what follows.

#### 2.2.1. Logistic Model

Based on a logistic regression analysis of patients treated by I-125 LDR, Zaider et al. [1] inferred the probability of unresolved Grade 2 or higher urethral toxicity at 12 months as a function of DU_20_:

(1) $$\text{NTC}{\text{P}}_{\text{ureth}}=P_{\text{tox},12}\left(\text{D}{\text{U}}_{20\text{-I}}\right)=\frac{1}{1+\exp\;\;\left[-\left(\gamma +\zeta {\text{DU}}_{20\text{-I}}\right)\right]},$$

where *γ* and *ζ* are two fitting parameters given with their uncertainties in Table 3. Further in the text, iodine-125 referring to the I-125 LDR equivalent implant will be noted I-125 whereas iodine-125 referring to our implants will be noted ^125^I.

#### 2.2.2. I-125 LDR Equivalent DU_20_

The parameters of the urethral NTCP model correspond to an I-125 LDR treatment. Hence, the DU_20_ for the different other radionuclides or mixtures has to be converted to an I-125 LDR equivalent DU_20_. This conversion will be referred to below as “I-125 conversion”. The Biologically Effective Dose (BED) is a powerful tool for this purpose as it allows a comparison of different treatment modalities.

The BED for an LDR implant whose seeds contain one radionuclide is [2, 25]

(2) $$\text{BE}{\text{D}}_{\text{LDR}}=\text{RB}{\text{E}}_{\max\;}\cdot \text{D}{\text{U}}_{20}+\frac{1}{\alpha /\beta }\frac{\lambda {\left(\text{D}{\text{U}}_{20}\right)}^{2}}{\left(\lambda +\mu \right)},$$

where RBE_max_ is the Relative Biological Effectiveness of the radionuclide at very low-dose rate; [2]; *α*/*β*  is the ratio of radiosensitivity parameters; *λ* is the radioactive decay constant of the radionuclide; *μ* is the sublethal cell damage repair rate. For the urethra, the commonly used values for *α*/*β*  and *μ* are 3 Gy and 0.5 h^−1^ respectively. For normal tissues, cell repopulation is not taken into account. The RBE_max_ values used in this study are from Wang et al. [26]: 1.41 for ^103^Pd and 1.28 for ^125^I. Due to the lack of experimental data on the RBE_max_ of ^131^Cs, its value has been set to the same value (i.e., 1.28) as that for ^125^I. This assumption is based on the fact that their emission spectra are similar. Note that (2) differs from the expression in Zaider et al. [1] one. However, as the sublethal cell damage repair rate for the urethra is much larger than the radioactive decay constant, both results fit within 1% in the worst case.

The clinical outcome would be equivalent for two treatments if they yield the same BED:

(3) $$\begin{array}{c} {\text{BED}}_{\text{LDR-}x}={\text{BED}}_{\text{LDR-I}} \end{array},$$

(4) $$\begin{array}{c} \begin{array}{c} \text{RB}{\text{E}}_{\text{max-}x}\cdot \text{D}{\text{U}}_{20\text{-}x}+\frac{1}{\alpha /\beta }\frac{\lambda_{x}{\left(\text{D}{\text{U}}_{20\text{-}x}\right)}^{2}}{\left(\lambda_{x}+\mu \right)}\\ \;\;\;=\text{RB}{\text{E}}_{\text{max-I}}\cdot \text{D}{\text{U}}_{20\text{-I}}+\frac{1}{\alpha /\beta }\frac{\lambda_{I}{\left(\text{D}{\text{U}}_{20\text{-I}}\right)}^{2}}{\left(\lambda_{\text{I}}+\mu \right)}. \end{array} \end{array}$$

The I-125 LDR equivalent DU_20-I_ of the DU_20-*x*_ resulting from an implant based on radionuclide *x* can be calculated by expressing (4) as a quadratic

(5) $$A{\left[\text{D}{\text{U}}_{20\text{-I}}\right]}^{2}+B\cdot \left[\text{D}{\text{U}}_{20\text{-I}}\right]+C=0,$$

where

(6) $$A=\frac{\lambda_{\text{I}}}{\left(\lambda_{\text{I}}+\mu \right)},$$

(7) $$B=\left(\frac{\alpha }{\beta }\right)\text{RB}{\text{E}}_{\max\;-\text{I}},$$

(8) $$C=-\left[\left(\frac{\alpha }{\beta }\right)\text{RB}{\text{E}}_{\max\;-x}\cdot \text{D}{\text{U}}_{20-x}+\frac{\lambda_{x}{\left(\text{D}{\text{U}}_{20-x}\right)}^{2}}{\left(\lambda_{x}+\mu \right)}\right].$$

As DU_20-I_, *A* and *B* must have positive values, the only valid solution of (5) is

(9) $$\text{D}{\text{U}}_{20\text{-I}}=\frac{-B+\sqrt{B^{2}-4AC}}{2A}.$$

For a bi-radionuclide seed implant, the solution is the same as (9) with same expression for *A* and *B*, but the expression of *C* is

(10) $$\begin{array}{cc} C & =-[\left(\frac{\alpha }{\beta }\right)\text{D}{\text{U}}_{20}\overset{2}{\underset{i=1}{\sum }}\delta_{i}\cdot \text{RB}{\text{E}}_{\text{max-}i}\\ & \;\;\;+2{\left(\text{D}{\text{U}}_{20}\right)}^{2}\overset{2}{\underset{i=1}{\sum }}\overset{2}{\underset{j=1}{\sum }}\frac{\lambda_{i}\lambda_{j}\delta_{i}\delta_{j}}{\left(\lambda_{i}+\mu \right)\left(\lambda_{i}+\lambda_{j}\right)}], \end{array}$$

where DU_20_ is the one of the bi-radionuclide implant and *δ*_*i*_ is the contribution of radionuclide *i* in the seed to the DU_20_.

#### 2.2.3. Bi-radionuclide Case: *δ*_*i*_ Contributions

Equation (10) requires that the contribution *δ*_*i*_ of each radionuclide to DU_20_ be known for each point in space that is considered. Treatment plans show that the relative contribution of each radionuclide can be considered as constant throughout the urethral volume. Hence, everywhere in the urethra:

(11) $$D=D_{1}+D_{2}=D\left(\delta_{1}+\delta_{2}\right)=D\left(\delta_{1}+\left(1-\delta_{1}\right)\right).$$

Let us include the RBE effect directly in the TPS file that defines the seed data (dose distribution in the adapted AAPM TG43U1 dosimetry formalism [23]). This can be done by modifying the adapted radial dose function and 1D-anisotropy function of the seed (see Appendix). The dose distribution *D*_RBE_ in the organs provided by the TPS would therefore also include the RBE effect:

(12) $$\begin{array}{cc} D_{\text{RBE}} & =D_{1}\text{RB}{\text{E}}_{\text{max-}1}+D_{2}\text{RB}{\text{E}}_{\text{max-}2},\\ & =D\left(\delta_{1}\text{RB}{\text{E}}_{\text{max-}1}+\delta_{2}\text{RB}{\text{E}}_{\text{max-}2}\right), \end{array}$$

(13) $$\begin{array}{cc} D_{\text{RBE}} & =D\left(\delta_{1}\text{RB}{\text{E}}_{\text{max-}1}+\left({1-\delta }_{1}\right)\text{RB}{\text{E}}_{\text{max-}2}\right). \end{array}$$

As the *δ*_*i*_'s are spatially independent, (13) can be applied directly to the DU_20_ value. Therefore, if the DU_20_ from the treatment plan with and without RBE effect (DU_20-RBE_ and DU_20_, resp.,) are known, then the contribution of each radionuclide to DU_20_ can be computed from

(14) $$\begin{array}{c} \delta_{1}=\frac{\left(\left(\text{D}{\text{U}}_{20\text{-RBE}}/\text{D}{\text{U}}_{20}\right)-\text{RB}{\text{E}}_{\text{max-}2}\right)}{\left(\text{RB}{\text{E}}_{\text{max-}1}-\text{RB}{\text{E}}_{\text{max-}2}\right)},\\ \delta_{2}=1-\delta_{1}. \end{array}$$

These values turn out to match within 1% the mean *δ*_*i*_'s that one would obtain at the middle of the urethra contour on each TRUS image slice. This result is not surprising as the urethra is approximately in the middle of the prostate and therefore in the middle of the seed distribution. All the seeds of the implant contribute with different magnitudes to urethral dose, providing a homogeneous dose distribution throughout the urethra for each radionuclide.

#### 2.2.4. Propagation of Uncertainty

The method described in the above three sections includes many parameters associated with uncertainties. The uncertainties in DU_20-I_ come from the radiobiological parameters (*μ*, *α*/*β*) and the RBE_max_ of each radionuclide. These uncertainties along with those of the NTCP fitting parameters will affect the urethral NTCP. The NTCP fitting parameters' uncertainties will not affect the relative NTCPs of the different seed implants as the fitting parameters are not radionuclide dependent. By contrast, DU_20-I_ parameters are radionuclide dependent. Therefore, the repercussion of the DU_20-I_ parameters' uncertainties on DU_20-I_ and the subsequent repercussion on urethral NTCP were studied.

The DU_20-I_ uncertainty can be expressed as follows:

(15) $$\begin{array}{cc} ∆{\text{DU}}_{20\text{-I}} & =\left|\frac{{\partial \text{DU}}_{20\text{-I}}}{\partial \mu }\right|∆\mu +\left|\frac{{\partial \text{DU}}_{20\text{-I}}}{\partial \left(\alpha /\beta \right)}\right|∆\left(\alpha /\beta \right)\\ & \;+\left|\frac{{\partial \text{DU}}_{20\text{-I}}}{\partial \delta_{1}}\right|{∆\delta }_{1}\\ & \;+|\frac{{\partial \text{DU}}_{20\text{-I}}}{\partial \text{RB}{\text{E}}_{\text{I-}125}}∆\text{RB}{\text{E}}_{\text{I-}125}+\frac{{\partial \text{DU}}_{20\text{-I}}}{\partial \text{RB}{\text{E}}_{\text{I}}}∆\text{RB}{\text{E}}_{1}\\ & \;\;+\frac{{\partial \text{DU}}_{20\text{-I}}}{\partial \text{RB}{\text{E}}_{2}}∆\text{RB}{\text{E}}_{2}|. \end{array}$$

The uncertainty on RBE values contains three different terms: the uncertainty on I-125 RBE and on the RBE of each radionuclide in the seed. Note that in the case of mono-radionuclide implants, ∂DU_20-I_/∂RBE_2_ = 0 and ∆*δ* = 0. If the implant uses ^125^I or ^131^Cs, their RBE_max_ value has to be the same as the I-125 RBE_max_ value for consistency reasons. RBE uncertainties are therefore correlated and the absolute value has to be taken over the whole RBE uncertainties. Moreover, the palladium-103 RBE is correlated to the iodine-125 RBE. Depending on the study [26, 27], if a high value of iodine-125 RBE_max_ (1.45 instead of 1.28) is chosen then a high value of palladium-103 RBE_max_ is obtained (1.75 instead of 1.41). As a result, the uncertainties on ^103^Pd RBE are correlated to the uncertainties on iodine-125 RBE, which can be rewritten as

(16) $$∆\text{RB}{\text{E}}_{\text{Pd}}=∆\text{RB}{\text{E}}_{\text{I}}\left(\frac{d\text{RB}{\text{E}}_{\text{Pd}}}{d\text{RB}{\text{E}}_{\text{I}}}\right).$$

The term into bracket has been calculated using the above mentioned value of ^103^Pd and ^125^I RBE_max_ values and is equal to 1.65.

The NTCP uncertainty expression includes only three terms:

(17) $$\begin{array}{cc} ∆\text{NTCP} & =\left|\frac{\partial \text{NTCP}}{\partial \gamma }\right|∆\gamma +\left|\frac{\partial \text{NTCP}}{\partial \zeta }\right|∆\zeta \\ & \;+\left|\frac{\partial \text{NTCP}}{\partial \text{D}{\text{U}}_{20\text{-I}}}\right|∆\text{D}{\text{U}}_{20\text{-I}}. \end{array}$$

The partial derivatives of DU_20-I_ and NTCP are given in Appendices B and C, respectively.

## 3. Results

### 3.1. Modeling Parameters and Associated Uncertainties

The radiobiological parameters used in this study are limited to the  *α*/*β* ratio and sublethal cell damage repair rate, *μ*. First, we choose the values that Zaider et al. used in their study. These are the one reported in Table 3. This high  *α*/*β* ratio is typical for tumors (typical range 5–25 Gy) but does not correspond to the commonly used value for late responding normal tissue (typical range 1–5 Gy) [2]. Therefore, we also calculate the DU_20-I_ and NTCP with *α*/*β* = 3 Gy (1–5 Gy) and *μ* = 0.5 h^−1^ (0–1.5 h^−1^) reported in the Dale and Jones text book [2]. The values into brackets are the uncertainty range used for the uncertainty calculations.

Relative Biological Effectiveness values are the one published by Wang et al. [26]. Due to the lack of experimental data on the RBE_max_ of ^131^Cs, its value has been set to the same value as the one of ^125^I. The uncertainty interval is based on RBE values published by other authors. The minimum RBE_max_ value is 1 by definition. The maximum RBE_max_ values have been set to the ones published by Antipas et al. [27]. 

Finally, the uncertainty on *δ*_1_, the contribution of the first radionuclide to the DU_20_, is equal to the maximal deviation with respect to the mean value of the *δ*_1_ obtained in the middle of the urethra on TRUS slices.

### 3.2. DU_20_ and I-125 LDR Equivalent DU_20_, DU_20-I_

**Figure 1:**
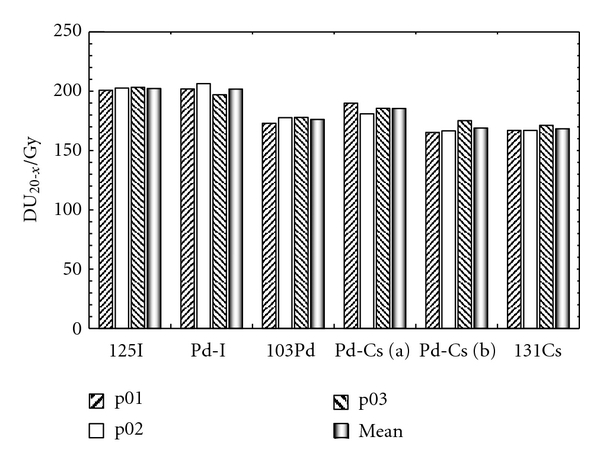
DU_20_ for the different seeds (mono- and bi-radionuclide) and patients. The DU_20_ averaged over the three patients is also plotted.

The TPS DU_20_ output for the different patients and radionuclides are presented in Figure 1. 

The mean DU_20_ value is also shown. As discussed above, these values are not comparable as they do not correspond to the same delivery scheme. Nevertheless, it will be interesting to observe how much the DU_20_ is modified for each seed implant after the I-125 conversion. It is emphasized that the DU_20_ does not change significantly from one patient to another one.

**Figure 2:**
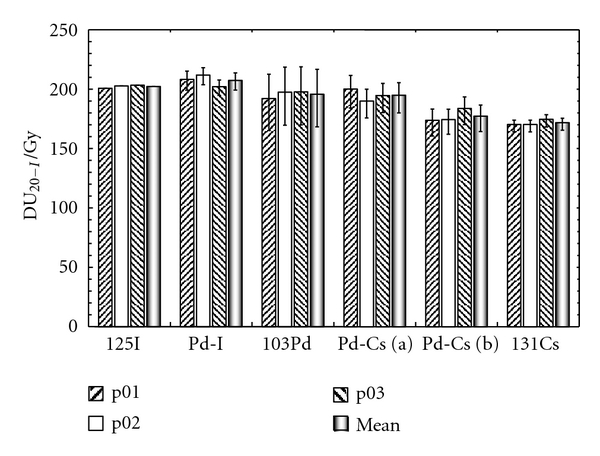
I-125 LDR equivalent DU_20-I_ results for the different implants (mono- and bi-radionuclide) and patients. The mean DU_20-I_ over the three patients is also plotted. Zaider et al. radiobiological parameters are used. Error bars were calculated using (15).

The implants can be compared after the I-125 conversion. The DU_20_ of each seed implant has been converted to an I-125 LDR equivalent DU_20_ using (6) to (10) and (14).  Figure 2 shows the results and the numerical values are reported in Table 4.

The effect of this conversion is to increase the DU_20_. The smallest increase is observed for the ^131^Cs implant with 3.3 Gy and the largest one for the ^103^Pd seed implant with 19.5 Gy. The uncertainty associated with these values will be the subject of a separate point. As expected, ^125^I is not affected by the I-125 conversion. The ^131^Cs implant is also not significantly affected by the I-125 conversion which suggests that the quadratic contribution of BED is small.

### 3.3. Urethral NTCP

**Figure 3:**
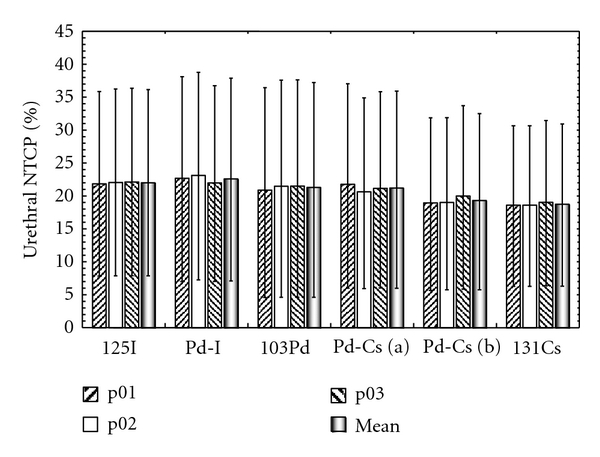
Urethral NTCP results for each patient and each implant (mono- and bi-radionuclide). The mean NTCP over the three patients is also plotted for each implant. Zaider et al. radiobiological parameters are used. Error bars were calculated using (17).

Although the DU_20-I_ figures correspond to the same delivery scheme (I-125 LDR) and they can be used to compare treatments, they do not give information about the probability of urethral complications. These probabilities have been computed using (1) and are plotted in Figure 3. Numerical values are reported in Table 4. 

The NTCPs are larger than the one published by Zaider et al. who obtained NTCPs of about 16% for 143 Gy I-125 LDR [1]. However, a more recent study by Zelefsky et al. reports 19% of patient experiencing late Grade 2 urinary symptoms [28]. 

All the NTCP results fall in the 19 to 23% range. The comparison between these data is therefore very difficult as these results are affected by large uncertainties. Their origin is discussed in the next section.

### 3.4. Uncertainties

We have calculated the contribution of each parameters uncertainty to the total DU_20-I_ and NTCP uncertainties. The values are tabulated in Tables 5 and 6, respectively.

Firstly, the results show that the DU_20-I_ uncertainty is mainly due to the uncertainty on the RBE_max_ value. The uncertainty is the greatest for ^103^Pd and the least for ^131^Cs apart from ^125^I which is not affected by the conversion and is not subjected to model-related errors. Sublethal damage repair rate, *μ*, as well as ratio *α*/*β*  have little effect on the DU_20-I_ value.

Secondly, the urethral NTCP uncertainties are mainly related to the parameter-fitting uncertainties. The error produced by ∆DU_20-I_ has a secondary importance. The total relative uncertainty is therefore almost constant among the different modalities, ranging from 12 to 17%.

**Figure 4:**
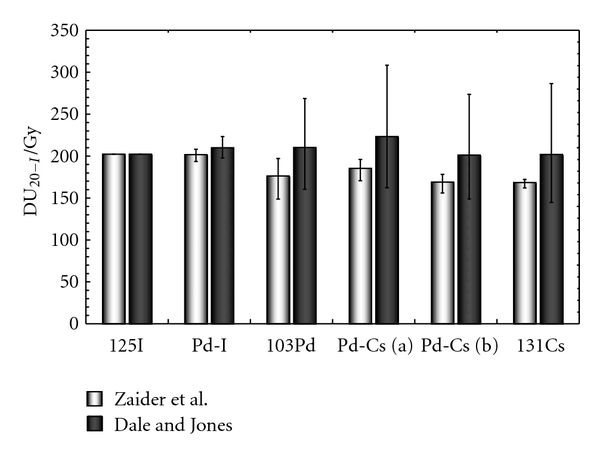
Comparison of DU_20−I_ for two different values of *α*/*β*  and *μ* : *α*/*β* = 16.6 Gy and *μ* = 1 h^−1^ (Zaider et al. [1]) and *α*/*β* = 3 Gy and *μ* = 0.5 h^−1^ (Dale and Jones [2]). Error bars were calculated using (15).

**Figure 5:**
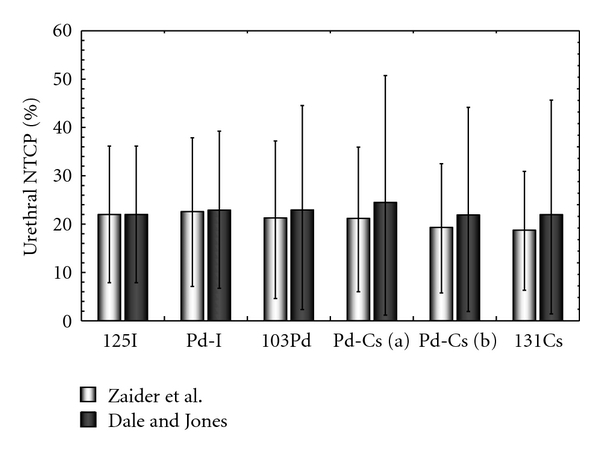
Comparison of urethral NTCP for two different values of *α*/*β*  and *μ* : *α*/*β* = 16.6 Gy and *μ* = 1 h^−1^ (Zaider et al. [1]) and *α*/*β* = 3 Gy and *μ* = 0.5 h^−1^ (Dale and Jones [2]). Error bars were calculated using (17).

However, these figures also depend on the radiobiological data chosen for the calculations. If the Dale and Jones radiobiological parameters (*α*/*β*  and *μ*) are used, the uncertainty generated by the sublethal damage repair rate becomes the most important one. The DU_20-I_ and urethral NTCP obtained with Zaider et al. and Dale and Jones radiobiological parameters are compared in Figures 4 and 5, respectively.

^131^Cs is the most affected radionuclide. RBE uncertainty remains almost unchanged. Uncertainties produced by *δ*_*i*_ contributions are still negligible compared to other parameter's uncertainties. These errors will have repercussions on NTCP uncertainties with the largest effect for ^131^Cs. The contribution of the total ∆DU_20-I_ on the NTCP total uncertainty is now of the same order of magnitude as the one produced by the parameter-fitting error.

## 4. Discussion and Conclusions

The variation in the DU_20_ due to I-125 conversion is influenced by two major contributions. First, the linear term in the BED expression depends only on the RBE_max_ value. Second, the quadratic term is more complex and results from the influence of three separate parameters: *α*/*β*  and *μ* which are tissue dependent and the radionuclide half-life.

Firstly, the ^125^I DU_20_ will not be affected by the I-125 conversion as only one radionuclide is involved. Secondly, as one chooses the same RBE_max_ value for ^131^Cs and ^125^I, the linear term of the ^131^Cs BED is not affected by the ^125^I conversion. Since the value of *α*/*β*  chosen by Zaider et al. is large, the quadratic contribution to the BED is small and influences the ^131^Cs BED by only 2%. If future experiments provide a RBE_max_ value for ^131^Cs that is different from that of ^125^I, larger variation in I-125 equivalent DU_20_ may be observed. Thirdly, ^103^Pd has a larger RBE_max_ value than ^125^I. Its linear contribution to BED will therefore also contribute to a modified value of the DU_20_. This contribution of the quadratic term accounts for only 1% of the total BED. The difference in RBE_max_ value between ^103^Pd and I-125 is therefore the main cause of the large increase in DU_20_ after I-125 conversion. Finally, the changes in the Pd-I and Pd-Cs DU_20_ due to I-125 conversion are related to both the linear and the quadratic contribution of each radionuclide to BED. Pd-Cs is the most affected mixture as both radionuclides differ from the one used for modeling (I-125).

The very similar values obtained for the NTCP and their large uncertainties makes it difficult, if not impossible, to conclude definitively whether, for equal tumour effect, bi-radionuclide brachytherapy would reduce the urethral complication probability relative to mono-radionuclide brachytherapy. Planning more patients would not improve the situation as these uncertainties are mainly due to the fitting parameter uncertainties of the empirical model of Zaider et al.

The large value assumed by Zaider et al. for the *α*/*β*  ratio reduces the influence of the quadratic term on the total BED considerably. Therefore the sublethal damage repair *μ* on the BED will show its effect only for radionuclides with short half-lives (like ^131^Cs). This is also proven by the low DU_20-I_ uncertainty associated with *μ* and to the *α*/*β*  ratio. The uncertainty related to the *δ*_1_ also indicates that our approximation will not significantly affect the final results.

However, the Zaider et al. radiobiological parameters are not the only ones in common use. The results obtained with the radiobiological parameters from Dale and Jones (Figures 4 and 5) clearly show that such parameters could also have a large impact on the final NTCP value. Moreover, the lower value of *α*/*β*  ratio increases the quadratic contribution of the BED, leading to larger uncertainties associated with *α*/*β*  and *μ*.

Finally, complication probabilities are certainly dependent on the way the treatment planning is performed. A logistic regression among patients treated by the each institution could also provide different NTCP parameter values. However, this would not affect the DU_20-I_ results.

We can conclude that the use of the urethra NTCP model for biologically based treatment planning in permanent seed prostate brachytherapy requires either better fitting parameters (with less associated errors) or a different NTCP model (different morbidity indicator than DU_20_ e.g.).

## Figures and Tables

**Table 1:** Prostate and urethra volumes for each planned patient.

Patient	p01	p02	p03
Prostate volume (cc)	24.64	38.9	45.33
Urethra volume (cc)	0.32	0.19	0.28

**Table 2:** Prescription dose for the different seeds used for planning.

	^103^Pd	^125^I	^131^Cs	Pd-I	Pd-Cs
Internal activity	100%	100%	100%	75%–25%	25%–75%
Prescription dose (Gy)	125	145	115	145	130 (a)
					115 (b)

**Table 3:** Modeling parameters and their absolute uncertainties. *p* is the value of the considered parameter.

Parameter	Units	*p*	*p* −∆	*p* +∆
*γ*	—	−2.60	−3.10	−2.10
*ζ*	Gy	0.0066	0.0050	0.0082
*μ*	h^−1^	1.0	0	1.5
*α*/*β*	Gy	16.6	5	25
RBE_*Pd*_	—	1.41	1.00	1.75
RBE_*I*_	—	1.28	1.00	1.45
RBE_*Cs*_	—	1.28	1.00	1.45
*δ*_1_	—	Variable	*δ*_1_ − 0.020	*δ*_1_ + 0.020

**Table 4:** Dose received by the hottest 20% of the urethra volume (DU_20_), 125I LDR equivalent DU_20_ (DU_20-I_), and urethra Normal Tissue Complication Probability (NTCP) for the different seed implants. The values are averaged over the three patients. Radiobiological parameters from Zaider et al. are used.

	DU_20_ (Gy)	DU_20-I_ (Gy)	NTCP (%)
^125^I	202.3	202.3	22.0
Pd-I	201.8	207.4	22.6
^103^Pd	176.3	195.8	21.3
Pd-Cs (a)	185.5	194.9	21.2
Pd-Cs (b)	169.0	177.3	19.3
^131^Cs	168.4	171.7	18.7

**Table 5:** DU_20-I_ uncertainty produced by each parameter and total DU_20-I_ uncertainty for each implant using Zaider et al. radiobiological parameters.

∆DU_20-I_ (Gy)		^125^I	Pd-I	^103^Pd	Pd-Cs (a)	Pd-Cs (b)	^131^Cs
*μ*	+∆	0.0	0.1	0.8	1.6	1.3	1.6
	−∆	0.0	0.3	1.6	3.1	2.6	3.3
*α*/*β*	+∆	0.0	0.1	0.8	1.6	1.3	1.7
	−∆	0.0	0.2	1.1	2.2	1.8	2.3
*δ*_1_	+∆	0.0	0.4	0.0	0.3	0.3	0.0
	−∆	0.0	0.4	0.0	0.3	0.3	0.0
RBE (tot)	+∆	0.0	5.6	19.3	7.1	6.4	0.4
	−∆	0.0	7.2	24.8	9.3	8.3	0.7

Total	+∆	0.0	6.3	20.9	10.6	9.3	3.7
	−∆	0.0	8.1	27.5	14.9	13.0	6.3

**Table 6:** NTCP uncertainty produced by each parameter and total NTCP uncertainty for each implant using Zaider et al. radiobiological parameters.

∆NTCP (%)		^125^I	Pd-I	^103^Pd	Pd-Cs (a)	Pd-Cs (b)	^131^Cs
*γ*	±∆	8.6%	8.7%	8.4%	8.4%	7.8%	7.6%
*ζ*	±∆	5.6%	5.8%	5.2%	5.2%	4.4%	4.2%
DU_20-I_	+∆	0.0%	0.7%	2.3%	1.2%	1.0%	0.4%
	−∆	0.0%	0.9%	3.0%	1.6%	1.3%	0.6%

Total	+∆	14.1%	15.3%	15.9%	14.7%	13.2%	12.2%
	−∆	14.1%	15.5%	16.7%	15.2%	13.6%	12.4%

## Appendix

### A. Inclusion of RBE in TPS Dose Distribution

In order to include the RBE effect in the TPS dose distribution, the radial dose function and the 1D or 2D anisotropy functions have to be modified. This modification is trivial in the case of mono-radionuclide brachytherapy: the radial dose function is multiplied by the radionuclide RBE_max_ value and the anisotropy functions are not altered. In the case of bi-radionuclide implants, the RBE_max_ value will be different from one radionuclide to another. Therefore,

(A.1) $$\begin{array}{cc} g_{\text{RBE}}\left(r\right) & =\overset{2}{\underset{i=1}{\sum }}c_{i}\text{RB}{\text{E}}_{\text{max-}i}g_{i}\left(r\right),\\ \phi_{\text{an-RBE}}\left(r\right) & =\frac{\sum_{i=1}^{2}c_{i}\text{RB}{\text{E}}_{\text{max-}i}g_{i}\left(r\right)\phi_{\text{an-}i}\left(r\right)}{\sum_{i=1}^{2}c_{i}\text{RB}{\text{E}}_{\text{max-}i}g_{i}\left(r\right)},\\ F\left(r,\theta \right) & =\frac{\sum_{i=1}^{2}c_{i}\text{RB}{\text{E}}_{\text{max-}i}g_{i}\left(r\right)F_{i}\left(r,\theta \right)}{\sum_{i=i}^{2}c_{i}\text{RB}{\text{E}}_{\text{max-}i}g_{i}\left(r\right)}. \end{array}$$

It can be shown that if (A.1) replaces (11), (14) and (16) in (4) or (5) of Nuttens and Lucas [23], this would give

(A.2) $$D\left(r,\theta \right)=\overset{2}{\underset{i=1}{\sum }}\text{RB}{\text{E}}_{\text{max-}i}D_{i}\left(r,\theta \right),$$

which means that the TPS dose distribution includes well the RBE effect.

### B. Partial Derivatives of DU_20-I_

DU_20-I_ is expressed as a function of *A*, *B*, and *C* ((6) to (10)). Its derivative with respect to parameter *p* can also be expressed as a function of *A*, *B*, and *C* and their derivative with respect to parameter *p* noted $(dA/dp)=\dot{A}$, $(dB/dp)=\dot{B}$ and $(dC/dp)=\dot{C}$

(B.1) $$\begin{array}{cc} \frac{d\text{D}{\text{U}}_{20\text{-I}}}{dp} & =\frac{1}{2A}\left(-\dot{B}+\frac{B\dot{B}-2\dot{A}C-2A\dot{C}}{\sqrt{B^{2}-4AC}}\right)\\ & \;-\frac{-B+\sqrt{B^{2}-4AC}}{2A^{2}}\dot{A}. \end{array}$$

The expressions of $\dot{A}$, $\dot{B}$, and $\dot{C}$ are now given for each parameter *p*.

(1) **p** = ***μ***,
  (B.2) $$\begin{array}{cc} & \dot{A}=\frac{dA}{d\mu }=\frac{-\lambda_{\text{I}}}{{\left(\lambda_{\text{I}}+\mu \right)}^{2}}=\frac{-A^{2}}{\lambda_{\text{I}}},\\ & \dot{B}=\frac{dB}{d\mu }=0,\\ & \dot{C}=\frac{dC}{d\mu }=2\overset{2}{\underset{i,j=1}{\sum }}\frac{\lambda_{i}\lambda_{j}\delta_{i}\delta_{j}{\text{DU}}_{20-x}^{2}}{{\left(\lambda_{i}+\mu \right)}^{2}\left(\lambda_{i}+\lambda_{j}\right)}, \end{array}$$
(2) **p** = (**α**/**β**),
  (B.3) $$\begin{array}{cc} & \dot{A}=\frac{dA}{d\left(\alpha /\beta \right)}=0,\\ & \dot{B}=\frac{dB}{d\left(\alpha /\beta \right)}=\text{RB}{\text{E}}_{\text{I-}125},\\ & \dot{C}=\frac{dC}{d\left(\alpha /\beta \right)}=-\text{D}{\text{U}}_{20-x}\overset{2}{\underset{i=1}{\sum }}\text{RB}{\text{E}}_{i}\delta_{i}, \end{array}$$
(3) **p** = **R** **B** **E**_I-125_,
  (B.4) $$\begin{array}{cc} & \dot{A}=\frac{dA}{d\text{RB}{\text{E}}_{\text{I-}125}}=0,\\ & \dot{B}=\frac{dB}{d\text{RB}{\text{E}}_{\text{I-}125}}=\frac{\alpha }{\beta },\\ & \dot{C}=\frac{dC}{d\text{RB}{\text{E}}_{\text{I-}125}}=0, \end{array}$$
(4) **p** = **R** **B** **E**_**i**_,
  (B.5) $$\begin{array}{cc} & \dot{A}=\frac{dA}{d\text{RB}{\text{E}}_{\text{i}}}=0,\\ & \dot{B}=\frac{dB}{d\text{RB}{\text{E}}_{\text{i}}}=0,\\ & \dot{C}=\frac{dC}{d\text{RB}{\text{E}}_{\text{i}}}=-\left(\frac{\alpha }{\beta }\right)\text{D}{\text{U}}_{20\text{-}x}\delta_{i}, \end{array}$$
(5) **p** = **δ**_1_,

These expressions apply only for the bi-radionuclide implant for which *δ*_1_ ≠ 1

(B.6) $$\begin{array}{cc} \dot{A} & =\frac{dA}{d\delta_{1}}=0 \end{array},$$

(B.7) $$\begin{array}{cc} \dot{B} & =\frac{dB}{d\delta_{1}}=0 \end{array},$$

(B.8) $$\begin{array}{cc} \dot{C} & =\frac{dC}{d\delta_{1}}\\ & =-\left(\frac{\alpha }{\beta }\right)\text{D}{\text{U}}_{20-x}\left(\text{RB}{\text{E}}_{1}-\text{RB}{\text{E}}_{2}\right)\\ & \;-2{\text{DU}}_{20-x}^{2}[\frac{\lambda_{1}\delta_{1}}{\left(\lambda_{1}+\mu \right)}+\frac{\lambda_{1}\lambda_{2}\left(1-2\delta_{1}\right)}{\left(\lambda_{1}+\mu \right)\left(\lambda_{1}+\lambda_{2}\right)}\\ & \;\;\;\;\;\;+\frac{\lambda_{1}\lambda_{2}\left(1-2\delta_{1}\right)}{\left(\lambda_{2}+\mu \right)\left(\lambda_{1}+\lambda_{2}\right)}+\frac{\lambda_{2}\left(\delta_{1}-1\right)}{\left(\lambda_{2}+\mu \right)}]. \end{array}$$

### C. Partial Derivatives of NTCP

The NTCP expression is given by (1) and its partial derivatives with respect to each parameter are

(C.1) $$\begin{array}{cc} & \frac{\partial \text{NTCP}}{\partial \gamma }=\frac{\exp\;\;\left[-\left(\gamma +\zeta \text{D}{\text{U}}_{20\text{-I}}\right)\right]}{{\left\{1+\exp\;\;\left[-\left(\gamma +\zeta \text{D}{\text{U}}_{20\text{-I}}\right)\right]\right\}}^{2}},\\ & \frac{\partial \text{NTCP}}{\partial \zeta }=\frac{\text{D}{\text{U}}_{20\text{-I}}\exp\;\;\left[-\left(\gamma +\zeta \text{D}{\text{U}}_{20\text{-I}}\right)\right]}{{\left\{1+\exp\;\;\left[-\left(\gamma +\zeta \text{D}{\text{U}}_{20\text{-I}}\right)\right]\right\}}^{2}},\\ & \frac{\partial \text{NTCP}}{\partial \text{D}{\text{U}}_{20\text{-I}}}=\frac{\zeta \exp\;\;\left[-\left(\gamma +\zeta \text{D}{\text{U}}_{20\text{-I}}\right)\right]}{{\left\{1+\exp\;\;\left[-\left(\gamma +\zeta \text{D}{\text{U}}_{20\text{-I}}\right)\right]\right\}}^{2}}. \end{array}$$
